# Use of natural anti-oxidants in experimental animal models of hepatic ischemia-reperfusion injury

**DOI:** 10.1016/j.amsu.2020.11.061

**Published:** 2020-11-26

**Authors:** Georgios Kyriakopoulos, Georgia Valsami, Christos Tsalikidis, Michail Pitiakoudis, Alexandra K. Tsaroucha

**Affiliations:** a Postgraduate Program in Hepatobiliary/Pancreatic Surgery, Faculty of Medicine, Democritus University of Thrace, Alexandroupolis, Greece; b School of Health Sciences, Department of Pharmacy, National and Kapodistrian University of Athens, Greece; c 2^nd^Department of Surgery, Faculty of Medicine, Democritus University of Thrace, Alexandroupolis, Greece; d Laboratory of Experimental Surgery, Faculty of Medicine, Democritus University of Thrace, Alexandroupolis, Greece

**Keywords:** Hepatic ischemia-reperfusion (I/R) injury, Oxidative stress, Anti-oxidants, Animal models, Ischemia duration

## Abstract

**Background:**

Ischemia-reperfusion injury (IRI) remains a clinical challenge in liver surgery, trauma and transplantation, contributing to morbidity and mortality worldwide. Thus, its impact, not only on the liver itself but also on remote tissues, has been studied during the last years. Different natural anti-oxidant substances have been researched in animal models, implementing different times of ischemia, aiming to test new therapeutic interventions.

**Objective:**

A literature review has been conducted with two goals: (1) to identify different natural anti-oxidants studied in experimental models; and (2) to summarize the various times of ischemia employed.

**Methods:**

Scientific papers published in PubMed for the period 2000–2020 were searched and reviewed.

**Results:**

More than 30 natural anti-oxidants have been tested. The time of ischemia ranged from 15 to 90 min with 60 min used most frequently, followed by 45 min. No studies were found with time exceeding 90 min.

**Conclusions:**

A significant number of research has been conducted on the use and protective effect of natural anti-oxidants in experimental animal models. Based on the published papers, 45–60 min seems to be the optimal duration of ischemia.

## Highlights

• Liver IRI is a multifactorial and complex process, involving many mechanisms, cells and mediators. Even though, most of these mechanisms have not been completely understood, several substances have been tested in experimental models in order to determine their protective or destructive role.
• Antioxidant therapy is a promising therapeutic pathway that can ameliorate the impact of liver ischemia-reperfusion injury.
• Non-pharmaceutical, natural extracts are increasingly gaining their place into the therapeutic options of physicians, in an attempt to avoid various adverse effects that the chemical drugs can cause.
• New unexplored research areas may include different strains of rats, more studies in larger mammals of comparable anatomy to humans, experiments on different liver diseases, publishing negative results regarding toxic doses of natural antioxidants, and testing different ischemia times.

## 1 Introduction

Ischemia-Reperfusion Injury (IRI) is a cascade of pathophysiological events in which cellular injury in oxygen-deprived tissue is paradoxically accentuated after oxygen delivery has been restored [1]. Its clinical applications can be seen not only during elective hepatectomies, but also during operations for management of liver trauma or in liver transplantation, where it determines the viability and functionality of the grafted organ [2].

Liver IRI is a multifactorial and complex process. During the ischemic phase, intracellular hypoxia affects the function of the mitochondrial respiratory chain, causing a significant imbalance to the reduction/oxidation status for which the mitochondrial enzymes are responsible. Thus, the oxidative phosphorylation process is inhibited and the intracellular stores of energy molecules are gradually depleted, mostly due to ADP and ATP consummation [3]. Consequently, the Na^+^/K^+^-ATPase pump is inhibited, and the cellular membranes start to become more permeable – sodium is accumulated into the cell, causing intracellular edema and finally death [4]. Calcium ions are also entering the cell, causing activation of the phospholipases of the cell membranes, leading to phospholipid degradation and cell membrane disruption [5]. High calcium concentrations degrade also the mitochondrial membranes, causing the release of cytochrome c (via the activation of the caspases system) and the further release of Reactive Oxygen Species (ROS) [6].

Hepatic IRI can be subdivided into cold IRI, which occurs during organ preservation prior to transplantation, and warm IRI (clinically associated with hypovolemic shock, hepatic surgery and liver transplantation). The main area of injury during cold ischemia are non-parenchymal cells (Kupffer cells - KC, Sinusoidal endothelial cells - SEC, Ito cells), whereas during warm ischemia the hepatocytes are mostly affected [7]. For the purpose of the current review, the discussion will be confined to warm IRI. Although ischemia and the subsequent hypoxia are significant stressors for every cell, the really severe damage is afflicted during the reperfusion phase.

Following reperfusion, the IRI starts developing. There are two distinct phases in that stage, the initial (or early or biochemical) phase and the late (inflammatory) phase. The former occurs <2 h after reperfusion starts. The main event is the activation of the complement (C5a) and the recruitment of CD4 T-cells [8], leading to the activation of the Kupffer cells, which are the resident macrophages of the liver [9]. Activated macrophages secrete the nuclear factor High Mobility Group Box 1(HMGB-1) as a cytokine mediator of inflammation [10]. The late phase occurs between 6 and 24 h after reperfusion. The central event now is the accumulation of ROS and cytokines such as Tumour Necrosis Factor–alpha (TNF-a) and Interleukin-1b (IL-1b) [11], leading to the transmigration of the activated neutrophils into the hepatic parenchyma and cellular injury and death via the pathways of necrosis (most commonly) [12] or apoptosis [13] and autophagy [14].

Reactive Oxygen Species– most commonly Hydrogen peroxide (H_2_O_2_) and hypochlorous acid – play a critical role in signal transduction pathways, coordinating the body's inflammatory response after hepatic IRI. They enhance pro-inflammatory gene expression (TNF-a, IL-1, IL-8 and cellular adhesion molecules) [15], induce the expression of NF-kB transcription factor [16], inactivate anti-proteases [17] and cause direct induction of necrotic and apoptotic cellular death [18].

TNF-a acts as the main mediator of the hepatic inflammatory response to IRI. The production of TNF-a causes the expression of ICAM (InterCellular Adhesion Molecule) and VCAM (Vascular Cell Adhesion Molecule) stimulating neutrophil activation and accumulation. The recruited and activated neutrophils release ROS and proteases that are responsible for the induced oxidative stress. Another event occurring during the reperfusion phase is the increased expression of Matrix Metalloproteinase-9 (MMP-9), a zinc-dependent enzyme that catalyzes the degradation of type IV collagen and gelatin.

Nitric Oxide Synthetase (NOS) catalyzes the formation of NO from l-arginine and it may have either cytotoxic or cytoprotective effects. This depends on the type of insult, the hepatic stored amounts of reduced glutathione, the amount duration of the NOS expression and the concomitant production of superoxide anions [19]. From its three isoforms, only two are expressed in the liver cells (eNOS and iNOS). eNOS expression is associated with reduced liver necrosis and acts as a potent liver sinusoidal vasodilator [20]. Even though the role of iNOS is still controversial, it probably contributes to reduced capillary perfusion and ultimately increased liver injury and increased mortality [21].

Heme Oxygenase-1(HO-1) is the rate-limiting enzyme that catalyzes the formation of CO, biliverdin and Fe^+2^ from heme degradation. Both CO and biliverdin are proven vasodilators and reduce necrosis/apoptosis as well [22]; thus, HO-1 has a protective role on hepatic IRI [23]. Studies have proven that the hepatocellular expression of HO-1 is up-regulated after IRI [24], reducing post-IRI hepatocellular apoptosis rate, increasing abundance of anti-oxidant substances and improving liver blood flow as well. All these mechanisms are shown to have a beneficial impact against IRI [25].

Multiple strategies have been proposed for the prevention of liver IRI or the reversal of its destructive effects. Local and remote ischemic pre-conditioning consists of improving solid organ resistance to IRI through intermittently interrupting the blood flow to a target organ or a site distant from the target organ. Similarly, ischemic post-conditioning (local and remote) refers to the protective effect of intermittent blood flow interruption, after reperfusion has occurred. Other strategies include natural anti-oxidants or artificial pharmacological agents, additives for preservation solutions, hypothermia, gene therapy and porto-caval shunt.

The main objective of this paper is to collect, review and summarize the existing literature, published from 2000 until today on anti-oxidant agents that have been studied in IRI experimental animal models. To the best of our knowledge there is no recent review on this topic.

## 2 Materials and methods

An extensive electronic search of the relevant literature was conducted using PubMed online database. The search included publications from January 2000 to January 2020. Animal experimental models, in which ischemia-reperfusion injury was studied, were searched. A combination of keyword searches was applied, as follows: Liver OR Hepatic – AND - Ischemia Reperfusion Injury OR IRI – AND - Animal model OR Experimental model OR Animal. The search was limited to full text articles published in English in peer-reviewed journals.

## 3 Results

The search yielded 4589 items, including 15 Systematic Reviews, 11 Randomized Control Trials (RCTs), 7 Meta-analyses and 425 Review Articles. All abstracts were printed and 2 Systematic Reviews, 2 RCTs, 1 Meta-analysis and 47 Review articles were short-listed, printed and meticulously reviewed. Thirteen more items were excluded for not referring to Ischemia Reperfusion Model. Finally, 39 original articles were included in the present review (Table 1).

**Table 1:** Natural anti-oxidants versus liver ischemia-reperfusion injury in animal models (2000–2020).

Study	Year of Publication	Species	Anti-oxidant substance	Dosage	Experimental model – Hepatic ischemia	Duration of hepatic ischemia (min)	Main parameters evaluated
Singh et al. [44]	2000	Rat	Picroliv	12 mg/kg	Total	30	caspase-3, FAS, MDA, IL-1a, IL-1b
Soltys et al. [31]	2001	Rat	Alpha-Tocopherol (Vitamin E) & sodium ascorbate	1000 u/kg/d	Partial	90	GSH, CAT, TOC, AA
Seo et al. [30]	2002	Rat	Ascorbic Acid (Vitamin C)	30, 100, 300 & 1000 mg/kg	Partial (Left liver only)	60	ALT, MDA, aminopyrine N-demethylase activity
Giakoustidis et al. [33]	2002	Rat	Alpha-Tocopherol (Vitamin E)	30–300 mg/kg	Total	60	AST, ALT, LDH, MDA
Glantzounis et al. [34]	2004	Rabbit	N-Acetylcysteine	150 mg/kg	Partial (Median & Left Lateral)	60	MAP, HR, Portal flow, ICG clearance, ALT, cytochrome oxidase
Smyrniotis et al. [35]	2005	Rat	N-Acetylcysteine	0.3 mg/g (300 mg/kg)	Partial (Median & Left Lateral)	60	AST, ALT, PLT aggregation, cAMP
Lee et al. [29]	2007	Rat	Ascorbic Acid (Vitamin C)	100 mg/kg	Partial (Left liver only)	90	AST, Glutathione, Glutamate dehydrogenase, Mitochondrial Malondialdehyde
Sehirli et al. [45]	2008	Rat	Grape Seed Extract	50 mg/kg	Total	45	AST, ALT, LDH, MDA, GSH, MPO, TNFa, IL1b
Liu et al. [46]	2008	Mouse	Apocynin & Allopurinol	3 mg/kg	Total	30	ALT, TNFa, GSH, MDA
Bauman et al. [37]	2008	Dog	N-Acetylcysteine	150 mg/kg	Total	60	ICG-PDR, AST, ALT
Keles et al. [36]	2008	Rat	N-Acetylcysteine & Ginkgo biloba extract	250 mg/kg	Partial (Left)	90	8- OhdG, ALT, AST, LDH thiobarbituric acid-reactive substance
Junnarkar et al. [47]	2009	Rat	Bucillamine	15 mg/kg/h	Partial (70%)	45	AST, ALT, RBC velocity, Sinusoidal perfusion, Leukocyte-Adherence molecules
Evans et al. [32]	2009	Mouse	Vitamin E succinate	50 IU/day	Total	15	ATP, GSH UCP2
Curek et al. [48]	2010	Rat	Astaxanthin	5 mg/kg/day	Partial (Median & Left Lateral)	60	ALT, XDH, XO, GSH, NOS2, nitrate
Rao et al. [49]	2010	Rat	All-trans Retinoic Acid	5–15 mg/kg/day	Partial (70%)	60	ALT, p38MARK, MDA, SOD
Kang et al. [38]	2011	Rat	Melatonin	10 mg/kg	Partial (Median & Left Lateral)	60	ALT, TLR3-TLR4, HMGB, MyD88, ERK, JNK, NF-kB, TRIF, IFN, IRF3, IFN-b, TNF-a, IL-6, iNOS, HO-1
Kim et al. [50]	2012	Mouse	Ferulic Acid	50, 100 & 200 mg/kg	Partial (Median & Left Lateral)	60	Caspase-3, cytochrome c, TNF-a, JNK1, JNK2
Lin et al. [51]	2012	Rat	Curcumin	25 mg/kg	Total	30	AST, ALT, LDH, MG, TNFa, NO, ATP
Nickkholgh et al. [52]	2012	Pig	Glutamine, antioxidants & green tea extract	70 g in 250 ml H_2_O	Total	40	HR, MAP, CVP, HAF, PVF, ALT, AST TNF-α, MPO, caspase-3
Yun et al. [53]	2012	Rat	Chlorogenic Acid	2.5, 5 & 10 mg/kg	Partial	60	ALT, TNFa, HO-1, HMGB1
Song et al. [55]	2012	Mouse	Grape Seed Proanthocyanidin	10, 20 & 40 mg/kg/day	Total	30	TNF-α, IL-1β l, T-AOC, MDA, SOD, CAT, GSH-PX, T-NOS, iNOS
Kireev et al. [39]	2013	Rat	Melatonin	10 mg/kg	Partial	35	ALT, AST, ATP, MDA, Nox metabolites, caspase-9, iNOS, eNOS, Bcl 2, Bax, Bad, AIF
Jiang et al. [56]	2013	Rat	Ambroxol	20, 80 & 140 mg/kg	Total	30	AST, ALT, SOD, CAT, GSH, MDA, Bcl-2, Bax, Caspase-3, JNK
Tsalkidou et al. [57]	2014	Rat	Apigenin	15 mg/kg	Total	45	TUNEL, Caspase 3, FAS/FASl
Tao et al. [59]	2014	Rat	Dioscin	20, 40 & 60 mg/kg	Partial (70%)	60	AST, ALT, SOD, CAT, GSH-Px, GSH, MDA, TNOS, iNOS, NO, IL1A, IL-6, TNFa, MIP-1, MIP-2, Fas, FasL, NF-JB, AP-1, COX-2, HMGB-1, CYP2E1, Bak, caspase-3, p53, PARP, Caspase-9, JNK, ERK-p38 MAPKs, Bcl-2, Bcl-x
Ramalho et al. [60]	2014	Rat	Rosmarinic acid	150 mg/kg	Partial (Median & Left Lateral)	60	AST, ALT, neutrophil accumulation, GSH, lipid hydroperoxide and nitrotyrosine, eNOS/iNOS, NO, TNF-α, Il-1b, NF-κB
Suyavaran et al. [61]	2015	Rat	Glutathione (GSH)	200 mg/kg	Partial (Median & Left Lateral)	90	AST, ALT, TNFa, active caspase-3 and PARP-1
Xu et al. [54]	2015	Rat	Grape Seed Proanthocyanidin	100 mg/kg/day	Partial (70%)	60	AST, ALT, glucose regulated protein 78, CCAAT, activating transcription factor-4, inositol-requiring enzyme-1, procaspase-12
Tao et al. [64]	2016	Mouse	Tea polyphenols	50 mg/kg	Partial (Left)	30	AST, ALT, Caspase-3, GSH/GSGG, iNOS
Sun et al. [40]	2017	Rat	Melatonin	20 & 50 mg/kg	Partial (Left)	60	MIF, MMP-9, IL-1β, TNF-α, COX-2, NOX-1, NOX-2, caspase 3 and PARP, ICAM-1, IL-1β, MMP-9, TNF-α, NF-κB
Mard et al. [62]	2017	Rat	Crocin	200 mg/kg	Partial (70%)	45	ALT, AST, ALP, SOD, CAT, bilirubin, p53
Uylas et al. [63]	2018	Rat	Quercetin	25, 50 & 100 mg/kg	Total	60	AST, ALT, MDA
Kyriakopoulos et al. [42]	2018	Rat	Silibinin		Total	45	TNF-α, M30
Tsaroucha et al. [41]	2018	Rat	Silibinin		Total	45	Fas/FasL, HMGB1, CD45
Tsaroucha et al. [58]	2016	Rat	Apigenin	5 mg	Total	45	BCL-2/BAX expression, M30/M65, ICAM-1
Michalinos et al. [43]	2020	Rat	Silibinin		Total	45	GPNMB expression

We recorded the natural antioxidant substance used to prevent hepatic IRI, some of the measured parameters and biomarkers as well as the duration and type (partial or total) of hepatic ischemia to the animal model.

## 4 Discussion

Liver tissue injury following reperfusion after a period of ischemia was first described in the middle 1970s in transplantation-related procedures [26]. We now know that IRI can occur in several other conditions, the most important being hypovolemic shock, mostly after hepatic surgery. Liver IRI is a multifactorial and complex process, involving many mechanisms, cells and mediators. Even though, most of these mechanisms have not been completely understood, several substances have been tested in experimental models in order to determine their protective or destructive role. The current study, reviews the existing literature and knowledge about antioxidant agents with protective or potentially protective effects against liver IR-injury.

Furthermore, the severity of hepatocellular damage is dependent not only on the degree of the blood flow occlusion applied, i.e., standard total Pringle manoeuvre versus partial ischemia via selective interruption of blood inflow, but also on the hepatic ischemia duration, which is the main focus of the present study. Several time-related experimental animal models (mostly in rodents) have been published. The time of occlusion varied between studies from 15 to 90 min, when no study applying ischemia for more than 90 min have been found in the reviewed literature. 60 min of ischemia (total or partial) is by far the most preferred ischemia duration, followed by 45 min. Almost half of studies (n = 15, 44%) report 60 min of ischemia, and the majority of the rest ranged between 30 and 45 min (n = 14, 41%). Only in four studies the ischemia time reached the 90 min limit and in one study the time was 15 min. As expected, all four studies that reported the outlier value of 90 min ischemia, applied exclusively partially ischemia.

As for the preferred technique, researchers applied to the animal model either the standard total Pringle manoeuvre for a certain duration or partial ischemia via selective interruption of blood inflow. Initially, we thought that total ischemia via Pringle manoeuvre would be the only method used, due to its technical feasibility. However, many authors have a preference to partial or lobar ischemia. This is induced by clamping the vascular pedicles of the median and/or left hepatic lobes, using an atraumatic microvascular clip, a process that cannot be achieved without the use of surgical loupes for proper magnification of the exact anatomical structures. This method produces a significant ischemic impact on the liver, without mesenteric venous congestion, that somebody would expect after a total inflow occlusion.

Another major point to underline is the percentage of the induced hepatic ischemia. Some of the studies caused total ischemia (n = 14, 41%), when the rest of them caused partial (n = 20, 59%). Regardless of the mechanism, partial ischemia involved the left lobe of the liver in most cases (n = 12, 5 exclusively the left liver and 7 both median and left lateral lobe). In five studies, investigators caused 70% ischemia of the liver parenchyma, whereas in three studies the allocation of ischemia was not reported.

Different species have been used in the experimental models, ranging from mice to pigs, in an attempt to escalate the resemblance to human application. The choice of the animal model depends essentially on the research question that is to be investigated. The vast majority of studies used rats (n = 26, 76%) and mice (n = 5, 15%), with the exception of three studies which selected a different type of animal (rabbit, dog and pig).

Most of research projects use smaller animals (usually rodents - mice or rats) due to availability of genetically manipulated animals and also due to the existence of reagents. The downside of these models is their “distance” from their human equivalents (different liver anatomy and size, faster metabolism), so there are significant limitations regarding their “translation” into the clinical setting for humans. Rodent models will always be the first acceptable step, but positive results can be escorted by studies performed in larger mammals which approach the human anatomy before being tried in proper clinical setting.

All research teams pay attention in selecting animals of the same gender (mostly male), probably due to sex-dependent factors (e.g., X-chromosome and variation in female sex hormone levels during the menstrual cycle) in a methodological attempt to avoid a selection bias error that could affect the standardization of the experiment. However, a recent meta-analysis has suggested that female rats are not more variable than male rats [27], so inclusion of both sexes in future studies is necessary to identify potential molecular mechanisms in a more accurate way. Researchers also try to choose animals of the same race and of similar age and body weight.

Natural anti-oxidants are very promising in IRI. The literature review revealed that most of the studies that look into the benefits of a substance derived from natural herb extracts come from scientific teams of Chinese or Middle Eastern/Mediterranean origin, areas that have been distinguished throughout generations with a rich inventory of natural medicinal herbs and where traditional medicine remains even nowadays a vibrant part of the health care system [28]. Western medicine has been criticizing these practices as controversial and fired some heated debate in health circles. However, during the last decades, western health researchers have increasingly started to adopt and look into the benefits of natural extracts, with the study of anti-oxidant substances being on the top of this list. We present the most important substances found the literature.

Two studies (27,28) tried to reveal a possible protective mechanism of ascorbic acid (AA). They were both in rats and caused partial ischemia for 60 and 90 min, respectively. Lee et al. concluded that AA can reduce the level of mitochondrial damage during I/R, increasing liver tolerance to reperfusion insult [29], while Seo et al. proved that AA acts as antioxidant at low doses whereas the pro-oxidant effects of ascorbic acid predominate at high doses [30].

The relatively low cost and toxicity profile of α-Tocopherol/Vitamin E makes it a very attractive therapeutic agent. Soltys et al. reported improved survival following ischemia in fatty livers [31], while eight years later Evans et al. concluded in increased viability in steatotic livers [32]. Giakoustidis et al. showed improvement of hepatic biochemistry after pre-treatment with α-tocopherol [33]. Four studies examined the possible effect of N-acetylcysteine (NAC) in hepatic ischemia [[34], [35], [36], [37]] with three of them supporting its protective role and one [37] that failed to confirm it.

We recorded 3 studies referring to melatonin [[38], [39], [40]], as it is believed to ameliorate I/R-induced liver damage by modulation of TLR-mediated inflammatory responses [38,39]. Recently, Sun et al. concluded that the results of their translational study provided important information on the therapeutic potential of xenogeneic ADMSC-derived exosomes in the treatment of acute IRI injury and underscore an additive protective effect through combining exosomes with melatonin in this experimental setting [40].

Silibinin (SLB) is a natural flavonoid, extract from the milk thistle seeds, known as the main ingredient of the antidote used against *Amanita phalloides* mushroom poisoning. The hepatoprotective and anti-inflammatory effect of the antioxidant agent silibinin was demonstrated through the reduction of the expression of specific biomarkers, i.e., Fas/FasL, HMGB-1, CD45, in liver tissue under IRI conditions by Tsaroucha et al. [41], when in a similar experimental protocol Kyriakopoulos et al. demonstrated its nephroprotective effect [42]. Michalinos et al. found an overexpression of glycoprotein non-metastatic melanoma B (GPNMB), in both the liver and the kidneys after hepatic IRI. Moreover, they confirmed the protective action of silibinin in liver injury through significant decrease in GPNMB expression [43].

Singh et al. looked into picroliv (a novel agent with antioxidant properties) and proved that it can attenuate warm hepatic ischemia–reperfusion injury in an in-vivo rat model of total hepatic ischemia for 30 min [44]. Pretreatment of rats with oral picroliv prior to induction of ischemia led to a reduced hepatocyte damage and preserved hepatocyte vitality.

Grape Seed Extract (GSE) is a natural antioxidant studied by Sehirli et al. [45]. The results clearly demonstrated that temporary blockade of hepatic blood supply yielded structural and functional alterations in the liver with a concomitant increase in proinflammatory cytokines in the blood. On the other hand, GSE treatment improved I/R-induced impairment in the liver functions, significantly decreased I/R-induced elevations in hepatic lipid peroxidation, myeloperoxidase activity and plasma cytokines, while decreased GSH levels were replenished by GSE treatment. These protective effects of grape seed extract on reperfusion-induced injury can be attributed, at least in part, to its ability to inhibit neutrophil infiltration, to balance oxidant–antioxidant status, and to regulate the generation of inflammatory mediators, suggesting a future role in the treatment of organ failures due to ischemia-reperfusion. This team used a rat model of total ischemia for 45 min.

Liu et al. studied apocynin (a natural organic compound structurally related to vanillin) and allopurinol, proving that pretreatment with both substances exerted protective effects on hepatic IRI [46]. The protection is associated with blocking the generation of superoxide anions during the hepatic I/R procedure, by inhibiting xanthine oxidase and NADPH oxidase activity and documented via the decrease of serum ALT, TNF-α, MDA contents and increased liver glutathione levels. This team used a mouse model of total ischemia for 30 min.

Bucillamine (a thiol derivative) was studied by Junnarkar et al. [47], showing that after pretreatment with this substance, the post-IRI serum AST was reduced, parenchymal blood flow was increased and the hepatocyte necrosis/apoptosis index decreased. This team used a rat model of partial (70%) ischemia for 45 min.

Curek et al. studied astaxanthin (ASX - a natural carotenoid) with the results suggesting that the ASX treatment can offer limited protection after liver IRI by reducing oxidant-induced protein carbonyl formation and conversion of XDH to XO [48]. The observed effect of ASX on liver enzymes, and oxidative stress was also reflected by a minor protection against histopathologic alterations. This team used a rat model of partial (median and left lateral lobe) ischemia of 60 min.

All-trans retinoic acid (atRA), an active metabolite of vitamin A with antioxidant effects, was studied by Rao et al. who demonstrated that pretreatment with atRA can attenuate the impact from IRI, by increasing MnSOD, which is associated with an increased activity of p38MAPK and Akt [49]. Thus, atRA has therapeutic potential in the prevention of IRI which could be tested in a clinical trial in the future. This study used a rat model of 70% partial liver ischemia for 60 min.

Kim et al. studied ferulic acid (FA), an organic phytochemical compound, proving that it protects hepatocytes against IRI through reduction of oxidative damage and JNK-mediated apoptotic signaling pathways [50]. This team used a mouse model of partial (median and left lateral) ischemia for 60 min.

Curcumin (a natural substance that is the principal curcuminoid ingredient of turmeric) was studied by Lin et al. [51]. Their experiments indicated that curcumin exerts protective effects on the reperfusion liver injury by attenuating the oxidative stress and inflammatory responses. Specifically, pretreatment with curcumin (25 mg/kg) significantly attenuated reperfusion liver injury, while the ATP content reversed. In addition, MG, TNF-α, and NO release were attenuated. This team used a rat model of total ischemia for 30 min.

Nickkholgh et al. studied the effects of an oral supplement containing glutamine, antioxidants and green tea extract (pONS) on a pig model of total ischemia with duration of 40 min [52]. They concluded that pONS significantly increased bile flow 8 h after reperfusion. ALT and AST were significantly lower after pONS administration. pONS significantly decreased the index of immunohistochemical expression for TNF-α, MPO, and cleaved caspase-3 proving that in general, administration of pONS before and after tissue damage protects the liver from warm IRI via mechanisms including decreasing oxidative stress, lipid peroxidation, apoptosis, and necrosis.

Yun et al. studied chlorogenic acid (CGA), suggesting that CGA protects against hepatic IRI [53]. The mechanism of action of CGA appears to involve its ability to inhibit HMGB1 release into the extracellular milieu, TLR4 overexpression, nuclear translocation of NF-κB and IRF-1 and pro-inflammatory mediator expression and to induce the Nrf2/HO-1 pathway. This team used a rat model of partial ischemia for 60 min.

Xu et al. studied the properties of grape seed proanthocyanidin (GSP), demonstrating that it possesses antioxidative, anti-inflammatory, and antiapoptotic effects by relieving ER stress to achieve a protection against liver IR [54]. This protective mechanism may be a result of the botanical ingredients that comprise GSP. This team used a rat model of partial (70%) ischemia for 60 min. The same substance (GSP) was studied by Song et al. with their data showing that the mechanism underlying the protection by GSP against hepatic IRI is associated with its ability of improving the oxidation resistance and inhibiting pro-inflammatory cytokines release, as well as augmenting the hypoxia tolerance responses [55]. In high-fat diet induced obese mice suffering hepatic IRI, GSP also showed its powerful ability on protecting hepatocyte function and decreasing the damage of warm hepatic IRI. This team studied a mouse model of total ischemia for 30 min.

Αmbroxol (a substance with mucolytic activity) was studied by Jiang et al. [56], who proved that pre-treatment with that molecule, reduced the histologic injury and significantly decreased serum ALT and AST levels, enhanced the activity of hepatic tissue SOD and CAT, increasing GSH but decreasing MDA tissue contents. On top of that, in the ambroxol group, Bcl-2 expression was increased and Bax and caspase-3 decreased compared with the control group. Furthermore, ambroxol reduced levels of phosphorylated JNK proving in conclusion that it can offer enhanced antioxidant and anti-apoptotic activities. This team used a rat model of total liver ischemia for 30 min.

Apigenin is a natural flavonoid that has shown hepatoprotective effects. Tsalkidou et al. looked into the potential benefits of apigenin and showed that its effects on the Fas/FasL mediated apoptotic pathway increased the expression of Fas gene in hepatocytes during the reperfusion phase and at the same time (4 h after reperfusion), apigenin reduced the concentration of Fas receptor [57]. The authors used a rat model of total liver ischemia for 45 min. The same team investigated possible protective effects of apigenin against IRI by measuring the expression of apoptosis controlling genes BCL-2 and BAX. Their findings suggest that apigenin has potent actions against hepatic IRI through suppression of inflammation, oxidative stress and inhibition of the process of apoptosis [58].

Tao et al. looked into the antioxidant properties of dioscin (a natural plant extract) and found that it significantly decreased serum ALT and AST activities, increased survival rate of rats, and improved I/R-induced hepatocyte abnormality [59]. In addition, dioscin increased the levels of SOD, CAT, GSH-Px, GSH, decreased the levels of MDA, TNOS, iNOS, NO, and prevented DNA fragmentation caused by IRI. Further research indicated that dioscin markedly decreased the gene expressions of interleukin-1A, interleukin-6, tumor necrosis factor-α, intercellular adhesion molecule-1, MIP-1, MIP-2, Fas, FasL, decreased the protein expressions of NF-JB, AP-1, COX-2, HMGB-1, CYP2E1, Bak, caspase-3, p53, PARP, Caspase-9, decreased the levels of JNK, ERK and p38 MAPKs phosphorylation, and upregulated the levels of Bcl-2 and Bcl-x. This team used a rat model of partial (70%) ischemia for 60 min.

Rosmarinic acid (RosmA), a natural compound found in many plants, was studied by Ramalho et al. in a rat model of partial 60 min ischemia [60]. Their team concluded that RosmA protects liver parenchymal cells against normothermic IRI by means of its vigorous anti-inflammatory and antioxidant properties. The mechanisms underlying these effects may be related to the inhibitory potential of RosmA on the NF-κB signaling pathway and to the reduction in hepatic iNOS and eNOS expressions and NO levels, in addition to its natural antioxidant capability, culminating with attenuation of the acute inflammatory response and that of oxidative/nitrosative stress following normothermic I/R in the liver.

Suyavaran et al. studied the effects of Glutathione (GSH) and their results revealed that the administration of GSH prior to hepatic I/R surgery protects both the young and aged rats from I/R-induced oxidative damage [61]. The pre-treatment with GSH significantly reduced the apoptosis and TNF-a by restoration of GSH/GSSG ratio at mitochondrial level, in young and aged rats. This team used a rat model of partial (median and left lateral) ischemia for 90 min.

Mard et al. studied crocin and zinc sulfate, with their results showing that Cr and ZnSO_4_, and their combined use protected the rat liver against I/R-induced hepatic injury through: (1) up-regulating the protein expression of Nrf2; (2) decreasing serum levels of miR-122 and miR-34a; (3) decreasing serum levels of ALT, AST and ALP; (4) increasing the antioxidant activity; and (5) reducing the protein expression of p53 following hepatic IR-induced injury. This team used a rat model of partial (70%) ischemia for 60 min [62].

Finally, quercetin (a natural plant extract from the flavonoid group of polyphenols) was studied by Uylas et al. in a rat model applying 60 min of total ischemia [63]. The authors concluded that quercetin can be effective in preventing hepatic IRI when the correct dose (50 mg/kg) was used. However, quercetin at 25 mg/kg dose was insufficient to protect the liver, while quercetin at 100 mg/kg dose showed adverse effects in the protection of the liver. They suggested that pretreatment with quercetin in a reliable dose may be effective to prevent the hepatic IRI during the hepatic vascular surgery or liver transplantation operations in clinical practice.

Our study has some limitations that need to be addressed. Firstly, the literature search was limited to articles published in English, and only one database (Pubmed). Secondly, it allows no direct comparison between antioxidants, given the small number of comparative studies published. Another methodological limitation is the inconsistency of the duration of hepatic ischemia, a decision made by authors with different criteria. Moreover, the experimental nature of the cited studies did not allow for large population samples, and, therefore, the possibility of underpowered analyses and other bias may exist. Finally, the lack of systematic and randomized clinical trials makes recommendations difficult.

## 5 Conclusion

Antioxidant therapy is a promising therapeutic pathway that can ameliorate the impact of liver ischemia-reperfusion injury. Non-pharmaceutical, natural extracts are increasingly gaining their place into the therapeutic options of physicians, in an attempt to avoid various adverse effects that the chemical drugs can cause. New unexplored research areas may include different strains of rats, more studies in larger mammals of comparable anatomy to humans, experiments on different liver diseases, publishing negative results regarding toxic doses of natural antioxidants, and testing different ischemia times.

## Declaration of competing interest

There is no conflict of interest.

## Abbreviations

(atRA): all-trans retinoic acid
A PI3K-Akt: Phosphatidylinositol 3-kinase
AA: Ascorbic acid
ALT: Alanine aminotransferase
AST: Aspartate aminotransferase
AX: Astaxanthin
cAMP: Cyclic adenosine monophosphate
CAT: Catalase
CGA: Chlorogenic acid
e-NOS: Endothelial NO synthase
FA: ferulic acid
GSE: Grape seed extract
GSH: Glutathione
GSH-PX: Glutathione peroxidase
GSP: Grape seed proanthocyanidin
(HO-1): Heme Oxygenase-1
HIF-1α: Hypoxia inducible factor 1-alpha
(HMGB-1): High Mobility Group Box 1
HO-1: Heme oxygenase-1
i-NOS: Inducible NO synthase
ICAM: (intercellular adhesion molecule)
ICAM-1: Intercellular adhesion molecule-1
(IL-1b): Interleukin-1b
IPC: Ischemic preconditioning
IPostC: Ischemic postconditioning
IR: Ischemia-reperfusion
IRI: Ischemia-reperfusion Injury
KC: Kupffer cells
LDH: Lactate dehydrogenase
MDA: Malondialdehyde
MIP-2: Macrophage inflammatory protein-2
mito-KATP: ATP-dependent mitochondrial potassium
MMP-9: Matrix Metalloproteinase-9
MnSOD: manganese superoxide dismutase
MPO: Myeloperoxidase
NF-κB: Nuclear factor-kappa beta
NO: Nitric oxide
NOS: Nitric Oxide Synthase
NVR: Necrotic volume ratio
pkc-ε: Protein kinase c-epsilon
RCT: Randomized Control Trials
rhEPO: Human recombinant erythropoietin
ROS: Reactive Oxygen Species
RT-PCR: Reverse transcriptase-polymerase chain reaction
SEC: Sinusoidal endothelial cells
SLB: Silibinin
SOD: Superoxide dismutase
TNF-α: Tumor necrosis factor-alpha
TOC: Tocopherol
TP: Tea Polyphenols
TUNEL: Terminal deoxynucleotidyl transferase dUTP nick end labeling
VCAM: vascular cells adhesion molecules)
VE: Vitamin E
VEGF: Vascular endothelial growth factor

## Research registration number

1. Name of the registry: N/A – It is an animal study.

2. Unique Identifying number or registration ID: N/A – It is an animal study.

3. Hyperlink to your specific registration (must be publicly accessible and will be checked): N/A – It is an animal study.

## References

[1] Jaeschke H. (2003). Molecular mechanisms of hepatic ischemia-reperfusion injury and preconditioning. Am. J. Physiol. Gastrointest. Liver Physiol..

[2] Clavien P.A., Harvey P.R., Strasberg S.M. (1992). Preservation and reperfusion injuries in liver allografts. An overview and synthesis of current studies. Transplantation.

[3] González-Flecha B., Cutrin J.C., Boveris A. (1993). Time course and mechanism of oxidative stress and tissue damage in rat liver subjected to in vivo ischemia-reperfusion. J. Clin. Invest..

[4] Blum H., Osbakken M.D., Johnson R.G. (1991). Sodium flux and bioenergetics in the ischemic rat liver. Magn. Reson. Med..

[5] Farber J.L. (1981). The role of calcium in cell death. Life Sci..

[6] Wang C., Youle R.J. (2009). The role of mitochondria in apoptosis*. Annu. Rev. Genet..

[7] Ikeda T., Yanaga K., Kishikawa K., Kakizoe S., Shimada M., Sugimachi K. (1992). Ischemic injury in liver transplantation: difference in injury sites between warm and cold ischemia in rats. Hepatology.

[8] Fondevila C., Busuttil R.W., Kupiec-Weglinski J.W. (2003). Hepatic ischemia/reperfusion injury--a fresh look. Exp. Mol. Pathol..

[9] Jaeschke H., Farhood A. (1991). Neutrophil and Kupffer cell-induced oxidant stress and ischemia-reperfusion injury in rat liver. Am. J. Physiol..

[10] Tsung A., Sahai R., Tanaka H., Nakao A., Fink M.P., Lotze M.T., Yang H., Li J., Tracey K.J., Geller D.A., Billiar T.R. (2005). The nuclear factor HMGB1 mediates hepatic injury after murine liver ischemia-reperfusion. J. Exp. Med..

[11] Clavien P.A., Harvey P.R., Sanabria J.R., Cywes R., Levy G.A., Strasberg S.M. (1993). Lymphocyte adherence in the reperfused rat liver: mechanisms and effects. Hepatology.

[12] Jaeschke H., Lemasters J.J. (2003). Apoptosis versus oncotic necrosis in hepatic ischemia/reperfusion injury. Gastroenterology.

[13] Gao W., Bentley R.C., Madden J.F., Clavien P.A. (1998). Apoptosis of sinusoidal endothelial cells is a critical mechanism of preservation injury in rat liver transplantation. Hepatology.

[14] Kim J.S., Nitta T., Mohuczy D., O'Malley K.A., Moldawer L.L., Dunn W.A., Behrns K.E. (2008). Impaired autophagy: a mechanism of mitochondrial dysfunction in anoxic rat hepatocytes. Hepatology.

[15] Liu T.Z., Lee K.T., Chern C.L., Cheng J.T., Stern A., Tsai L.Y. (2001). Free radical-triggered hepatic injury of experimental obstructive jaundice of rats involves overproduction of proinflammatory cytokines and enhanced activation of nuclear factor kappaB. Ann. Clin. Lab. Sci..

[16] Harada N., Iimuro Y., Nitta T., Yoshida M., Uchinami H., Nishio T., Hatano E., Yamamoto N., Yamamoto Y., Yamaoka Y. (2003). Inactivation of the small GTPase Rac 1 protects the liver from ischemia/reperfusion injury in the rat. Surgery.

[17] Jaeschke H. (2011). Reactive oxygen and mechanisms of inflammatory liver injury: present concepts. J. Gastroenterol. Hepatol..

[18] Rüdiger H.A., Clavien P.A. (2002). Tumor necrosis factor alpha, but not Fas, mediates hepatocellular apoptosis in the murine ischemic liver. Gastroenterology.

[19] Cuzzocrea S., Riley D.P., Caputi A.P., Salvemini D. (2001). Antioxidant therapy: a new pharmacological approach in shock, inflammation, and ischemia/reperfusion injury. Pharmacol. Rev..

[20] Lee V.G., Johnson M.L., Baust J., Laubach V.E., Watkins S.C., Billiar T.R. (2001). The roles of iNOS in liver ischemia-reperfusion injury. Shock.

[21] Kimura H., Katsuramaki T., Isobe M., Nagayama M., Meguro M., Kukita K., Nui A., Hirata K. (2003). Role of inducible nitric oxide synthase in pig liver transplantation. J. Surg. Res..

[22] Katori M., Anselmo D.M., Busuttil R.W., Kupiec-Weglinski J.W. (2002). A novel strategy against ischemia and reperfusion injury: cytoprotection with heme oxygenase system. Transpl. Immunol..

[23] Lv X., Yang L., Tao K., Liu Y., Yang T., Chen G., Yu W., Lv H., Wu F. (2011). Isoflurane preconditioning at clinically relevant doses induce protective effects of heme oxygenase-1 on hepatic ischemia reperfusion in rats. BMC Gastroenterol..

[24] Lai I.R., Chang K.J., Chen C.F., Tsai H.W. (2006). Transient limb ischemia induces remote preconditioning in liver among rats: the protective role of heme oxygenase-1. Transplantation.

[25] Origassa C.S., Câmara N.O. (2013). Cytoprotective role of heme oxygenase-1 and heme degradation derived end products in liver injury. World J. Hepatol..

[26] Galaris D., Barbouti A., Korantzopoulos P. (2006). Oxidative stress in hepatic ischemia-reperfusion injury: the role of antioxidants and iron chelating compounds. Curr. Pharmaceut. Des..

[27] Becker J.B., Prendergast B.J., Liang J.W. (2016). Female rats are not more variable than male rats: a meta-analysis of neuroscience studies. Biol. Sex Differ..

[28] Gu S., Pei J. (2017). Innovating Chinese herbal medicine: from traditional health practice to scientific drug discovery. Front. Pharmacol..

[29] Lee W.Y., Lee J.S., Lee S.M. (2007). Protective effects of combined ischemic preconditioning and ascorbic acid on mitochondrial injury in hepatic ischemia/reperfusion. J. Surg. Res..

[30] Seo M.Y., Lee S.M. (2002). Protective effect of low dose of ascorbic acid on hepatobiliary function in hepatic ischemia/reperfusion in rats. J. Hepatol..

[31] Soltys K., Dikdan G., Koneru B. (2001). Oxidative stress in fatty livers of obese Zucker rats: rapid amelioration and improved tolerance to warm ischemia with tocopherol. Hepatology.

[32] Evans Z.P., Mandavilli B.S., Ellett J.D., Rodwell D., Fariss M.W., Fiorini R.N., Schnellmann R.G., Schmidt M.G., Chavin K. (2009). Vitamin E succinate enhances steatotic liver energy status and prevents oxidative damage following ischemia/reperfusion. Transplant. Proc..

[33] Giakoustidis D., Papageorgiou G., Iliadis S., Kontos N., Kostopoulou E., Papachrestou A., Tsantilas D., Spyridis C., Takoudas D., Botsoglou N., Dimitriadou A., Giakoustidis E. (2002). Intramuscular administration of very high dose of alpha-tocopherol protects liver from severe ischemia/reperfusion injury. World J. Surg..

[34] Glantzounis G.K., Yang W., Koti R.S., Mikhailidis D.P., Seifalian A.M., Davidson B.R. (2004). Continuous infusion of N-acetylcysteine reduces liver warm ischaemia-reperfusion injury. Br. J. Surg..

[35] Smyrniotis V., Arkadopoulos N., Kostopanagiotou G., Theodoropoulos T., Theodoraki K., Farantos C., Kairi E., Paphiti A. (2005). Attenuation of ischemic injury by N-acetylcysteine preconditioning of the liver. J. Surg. Res..

[36] Keles M.S., Demirci N., Yildirim A., Atamanalp S.S., Altinkaynak K. (2008). Protective effects of N-acetylcysteine and Ginkgo biloba extract on ischaemia-reperfusion-induced hepatic DNA damage in rats. Clin. Exp. Med..

[37] Baumann J., Ghosh S., Szakmany T., Jancso G., Ferencz A., Roth E., Bogar L. (2008). Short-term effects of N-acetylcysteine and ischemic preconditioning in a canine model of hepatic ischemia-reperfusion injury. Eur. Surg. Res..

[38] Kang J.W., Koh E.J., Lee S.M. (2011). Melatonin protects liver against ischemia and reperfusion injury through inhibition of toll-like receptor signaling pathway. J. Pineal Res..

[39] Kireev R., Bitoun S., Cuesta S., Tejerina A., Ibarrola C., Moreno E., Vara E., Tresguerres J.A. (2013). Melatonin treatment protects liver of Zucker rats after ischemia/reperfusion by diminishing oxidative stress and apoptosis. Eur. J. Pharmacol..

[40] Sun C.K., Chen C.H., Chang C.L., Chiang H.J., Sung P.H., Chen K.H., Chen Y.L., Chen S.Y., Kao G.S., Chang H.W., Lee M.S., Yip H.K. (2017). Melatonin treatment enhances therapeutic effects of exosomes against acute liver ischemia-reperfusion injury. Am J Transl Res.

[41] Tsaroucha A.K., Valsami G., Kostomitsopoulos N., Lambropoulou M., Anagnostopoulos C., Christodoulou E., Falidas E., Betsou A., Pitiakoudis M., Simopoulos C.E. (2018). Silibinin effect on fas/FasL, HMGB1, and CD45 expressions in a rat model subjected to liver ischemia-reperfusion injury. J. Invest. Surg..

[42] Kyriakopoulos G., Tsaroucha A.K., Valsami G., Lambropoulou M., Kostomitsopoulos N., Christodoulou E., Kakazanis Z., Anagnostopoulos C., Tsalikidis C., Simopoulos C.E. (2018). Silibinin improves TNF-α and M30 expression and histological parameters in rat kidneys after hepatic ischemia/reperfusion. J. Invest. Surg..

[43] Michalinos A., Tsaroucha A.K., Lambropoulou M., Schizas D., Valsami G., Kostomitsopoulos N., Pitiakoudis M.S., Simopoulos C.E. (2020). Glycoprotein non-metastatic melanoma B expression after hepatic ischemia reperfusion and the effect of silibinin. Transl Gastroenterol Hepatol.

[44] Singh A.K., Mani H., Seth P., Gaddipati J.P., Kumari R., Banuadha K.K., Sharma S.C., Kulshreshtha D.K., Maheshwari R.K. (2000). Picroliv preconditioning protects the rat liver against ischemia-reperfusion injury. Eur. J. Pharmacol..

[45] Sehirli O., Ozel Y., Dulundu E., Topaloglu U., Ercan F., Sener G. (2008). Grape seed extract treatment reduces hepatic ischemia-reperfusion injury in rats. Phytother Res..

[46] Liu P.G., He S.Q., Zhang Y.H., Wu J. (2008). Protective effects of apocynin and allopurinol on ischemia/reperfusion-induced liver injury in mice. World J. Gastroenterol..

[47] Junnarkar S.P., Tapuria N., Dutt N., Fuller B., Seifalian A.M., Davidson B.R. (2009). Bucillamine improves hepatic microcirculation and reduces hepatocellular injury after liver warm ischaemia-reperfusion injury. HPB.

[48] Curek G.D., Cort A., Yucel G., Demir N., Ozturk S., Elpek G.O., Savas B., Aslan M. (2010). Effect of astaxanthin on hepatocellular injury following ischemia/reperfusion. Toxicology.

[49] Rao J., Zhang C., Wang P., Lu L., Zhang F. (2010). All-trans retinoic acid alleviates hepatic ischemia/reperfusion injury by enhancing manganese superoxide dismutase in rats. Biol. Pharm. Bull..

[50] Kim H.Y., Lee S.M. (2012). Ferulic acid attenuates ischemia/reperfusion-induced hepatocyte apoptosis via inhibition of JNK activation. Eur. J. Pharmaceut. Sci..

[51] Lin C.M., Lee J.F., Chiang L.L., Chen C.F., Wang D., Su C.L. (2012). The protective effect of curcumin on ischemia-reperfusion-induced liver injury. Transplant. Proc..

[52] Nickkholgh A., Li Z., Yi X., Mohr E., Liang R., Mikalauskas S., Gross M.L., Zorn M., Benzing S., Schneider H., Büchler M.W., Schemmer P. (2012). Effects of a preconditioning oral nutritional supplement on pig livers after warm ischemia. HPB Surg..

[53] Yun N., Kang J.W., Lee S.M. (2012). Protective effects of chlorogenic acid against ischemia/reperfusion injury in rat liver: molecular evidence of its antioxidant and anti-inflammatory properties. J. Nutr. Biochem..

[54] Xu Z.C., Yin J., Zhou B., Liu Y.T., Yu Y., Li G.Q. (2015). Grape seed proanthocyanidin protects liver against ischemia/reperfusion injury by attenuating endoplasmic reticulum stress. World J. Gastroenterol..

[55] Song X., Xu H., Feng Y., Li X., Lin M., Cao L. (2012). Protective effect of grape seed proanthocyanidins against liver ischemic reperfusion injury: particularly in diet-induced obese mice. Int. J. Biol. Sci..

[56] Jiang K., Wang X., Mao X., Lao H., Zhang J., Wang G., Cao Y., Tong I., Zhang F. (2013). Ambroxol alleviates hepatic ischemia reperfusion injury by antioxidant and antiapoptotic pathways. Transplant. Proc..

[57] Tsalkidou E.G., Tsaroucha A.K., Chatzaki E., Lambropoulou M., Papachristou F., Trypsianis G., Pitiakoudis M., Vaos G., Simopoulos C. (2014). The effects of apigenin on the expression of Fas/FasL apoptotic pathway in warm liver ischemia-reperfusion injury in rats. BioMed Res. Int..

[58] Tsaroucha A.K., Tsiaousidou A., Ouzounidis N., Tsalkidou E., Lambropoulou M., Giakoustidis D., Chatzaki E., Simopoulos C. (2016). Intraperitoneal administration of apigenin in liver ischemia/reperfusion injury protective effects. Saudi J. Gastroenterol..

[59] Tao X., Wan X., Xu Y., Xu L., Qi Y., Yin L., Han X., Lin Y., Peng J. (2014). Dioscin attenuates hepatic ischemia-reperfusion injury in rats through inhibition of oxidative-nitrative stress, inflammation and apoptosis. Transplantation.

[60] Ramalho L.N., Pasta Â A., Terra V.A., Augusto M., Sanches S.C., Souza-Neto F.P., Cecchini R., Gulin F., Ramalho F.S. (2014). Rosmarinic acid attenuates hepatic ischemia and reperfusion injury in rats. Food Chem. Toxicol..

[61] Suyavaran A., Ramamurthy C., Mareeswaran R., Subastri A., Lokeswara Rao P., Thirunavukkarasu C. (2015). TNF-α suppression by glutathione preconditioning attenuates hepatic ischemia reperfusion injury in young and aged rats. Inflamm. Res..

[62] Mard S.A., Akbari G., Dianat M., Mansouri E. (2017). Protective effects of crocin and zinc sulfate on hepatic ischemia-reperfusion injury in rats: a comparative experimental model study. Biomed. Pharmacother..

[63] Uylaş M.U., Şahin A., Şahintürk V., Alataş İ. (2018). Quercetin dose affects the fate of hepatic ischemia and reperfusion injury in rats: an experimental research. Int. J. Surg..

[64] Tao J., Shen X., Ai Y., Han X. (2016). Tea polyphenols protect against ischemia/reperfusion-induced liver injury in mice through anti-oxidative and anti-apoptotic properties. Exp Ther Med.

